# Mycorrhizal Fungal Diversity and Community Composition in Two Closely Related *Platanthera* (Orchidaceae) Species

**DOI:** 10.1371/journal.pone.0164108

**Published:** 2016-10-03

**Authors:** Fabiana Esposito, Hans Jacquemyn, Michael Waud, Daniel Tyteca

**Affiliations:** 1 Earth and Life Institute, Biodiversity Research Centre, Université catholique de Louvain, Croix du Sud 4-5 (L7.07.04), B-1348 Louvain-la-Neuve, Belgium; 2 Department of Biology, Plant Conservation and Population Biology, KULeuven, B-3001, Leuven, Belgium; University of Naples Federico II, ITALY

## Abstract

While it is generally acknowledged that orchid species rely on mycorrhizal fungi for completion of their life cycle, little is yet known about how mycorrhizal fungal diversity and community composition vary within and between closely related orchid taxa. In this study, we used 454 amplicon pyrosequencing to investigate variation in mycorrhizal communities between pure (allopatric) and mixed (sympatric) populations of two closely related *Platanthera* species (*Platanthera bifolia* and *P*. *chlorantha*) and putative hybrids. Consistent with previous research, the two species primarily associated primarily with members of the Ceratobasidiaceae and, to a lesser extent, with members of the Sebacinales and Tulasnellaceae. In addition, a large number of ectomycorrhizal fungi belonging to various families were observed. Although a considerable number of mycorrhizal fungi were common to both species, the fungal communities were significantly different between the two species. Individuals with intermediate morphology showed communities similar to *P*. *bifolia*, confirming previous results based on the genetic architecture and fragrance composition that putative hybrids essentially belonged to one of the parental species (*P*. *bifolia*). Differences in mycorrhizal communities between species were smaller in mixed populations than between pure populations, suggesting that variation in mycorrhizal communities was largely controlled by local environmental conditions. The small differences in mycorrhizal communities in mixed populations suggests that mycorrhizal fungi are most likely not directly involved in maintaining species boundaries between the two *Platanthera* species. However, seed germination experiments are needed to unambiguously assess the contribution of mycorrhizal divergence to reproductive isolation.

## Introduction

For completion of their life cycle, most orchid species rely on at least two biotic interactions that occur both below and above the ground [1]. First, the majority of orchid species rely on pollinators for successful fruit and seed set. Although fruit set in orchids is generally low [2,3], this is usually compensated by the vast number of seeds produced within a single fruit. However, due to their tiny size and the absence of endosperm (i.e. necessary nutritional resources within plant seeds) [4], orchid seeds have become completely dependent on mycorrhizal fungi for seed germination and successful establishment of seedlings [5,6]. As a result, it can be expected that the spatial distribution and abundance of orchids is to a large extent dependent on the contingencies of the spatial distribution of their mycorrhizal partners [7,8].

While it is clear that mycorrhizal fungi are an indispensable part of the life-history of orchids, little is known about how mycorrhizal communities differ between populations within species or between species within a genus. Recent research has shown that mycorrhizal communities can be highly variable between species or even between populations within species [9,10]. For example, populations of the terrestrial orchid *Neottia ovata* inhabiting forest and meadows showed significant differences in mycorrhizal communities between these habitats [11]. Recently diverged *Epipactis* species that occupy different habitats were also characterized by significantly different mycorrhizal communities [12]. Furthermore, in co-occurring orchid species, mycorrhizal interactions also tend to be species-specific with little sharing of mycorrhizal taxa between orchid species [8,10,13,14]. These findings suggest that there is a possibility that habitat-specific adaptations in general and mycorrhizal divergence in particular maintain species boundaries and mediate coexistence of multiple orchid species.

Although it is widely accepted that orchid species are often prone to shifts in pollinators, leading to ecotype formation [15], less is known about how mycorrhizae influence taxon formation. Nonetheless, it can be assumed that differences in mycorrhizal communities contribute to reproductive isolation acting between recently diverged sister species in at least two different ways. First, seed germination experiments have shown that in places where multiple orchids grow, which associate with different sets of mycorrhizal fungi, the seeds of one species did not germinate at sites where the other species grows and vice versa [16,17]. These results suggest that in orchid species that associate with strongly different mycorrhizal communities, seeds may fail to germinate in heterospecific habitat and that very few or no seedlings will establish (i.e. immigrant inviability) [18,19]. As a result, populations of the two species will occur in disparate geographical regions so that gene flow by pollen or seeds between habitats is restricted (i.e. ecogeographic isolation). Ultimately, this will directly reduce gene flow and the possibility of heterospecific mating and therefore contribute to reproductive isolation. Second, there is some evidence that hybrid seeds only germinate in the immediate vicinity of the maternal plant, but not of the paternal plant [20]. In this case, hybrid seeds are no longer compatible with the mycorrhizal fungi of one or both species and therefore suffer reduced germination and/or survival compared to parental seeds (F_1_ inviability).

In this study, we investigated natural variation in mycorrhizal communities associating with two closely related *Platanthera* species (*Platanthera bifolia* (L.) L.C. Rich. and *P*. *chlorantha* (Cust.) Rchb). Previous research has shown that in pure (allopatric) populations the two species exhibit significant morphological, phenological and ecological differences that should prevent interspecific gene flow and hybridization [21]. However, when the two species co-occur (sympatric populations), individuals showing intermediate morphological characters have often been found [22–26], suggesting that the species hybridize occasionally under natural conditions and that the observed morphological and phenological differences are not sufficient to maintain species boundaries. On the other hand, detailed analyses of the genetic architecture and floral scent in two mixed populations in Belgium, where these typical intermediate individuals co-occurred with the two parental *Platanthera* species, showed that plants with intermediate morphological characters essentially belonged to *P*. *bifolia* and that true hybrids were extremely rare. Moreover, these individuals with intermediate characteristics were spatially distributed in close vicinity of *P*. *bifolia* plants [27]. These results suggest that hybridization between both species is restricted and that the mycorrhizal communities associating with the two species may be significantly different and that intermediate individuals have mycorrhizal communities similar to those associating with *P*. *bifolia*.

To test these hypotheses, we investigated variation in mycorrhizal communities associating with *P*. *bifolia*, *P*. *chlorantha* and intermediate individuals. Since local environmental conditions have been shown to strongly impact on the variability of mycorrhizal communities [9,10], sampling was conducted in a diverse set of populations, including both pure (allopatric) and mixed (sympatric) populations. We further hypothesized that if mycorrhizal fungi are involved in maintaining species boundaries between the two *Platanthera* species, mycorrhizal divergence in mixed populations should be as large as or larger than in pure populations. In contrast, when differences in local environmental conditions are more important in determining mycorrhizal communities, mixed populations should display higher similarities in mycorrhizal communities than pure populations.

## Materials and Methods

### Study Populations

*Platanthera bifolia* and *P*. *chlorantha* are two terrestrial orchids that have a wide Eurasian distribution [28]. The two species belong to the orchid genus *Platanthera*, which can be considered as the most represented terrestrial orchid genus in the Old World with about 85 species [29–33]. Species within the genus *Platanthera* are mainly pollinated by Lepidoptera, most often moths, which are attracted to the flower by the white colour and the strong, heavy scent that is emitted early in the evening or during the night [34,35]. In Europe, *P*. *bifolia* and *P*. *chlorantha* flower between May and July, and there is some overlap in flowering period in areas of sympatry [36].

Both species display 10–25 white hermaphroditic flowers, which open sequentially, basically to apically, and which have a slender, long nectariferous spur as a backward extension of the lip petal. The morphology of the column, and more in particular the distance between the viscidia, differs strongly between the two species [21,37]. In *P*. *bifolia*, the viscidia are close to each other and the anther pockets stand almost parallel. The species is predominantly pollinated by moths of the Sphingidae, which attach the pollinia on the proboscis [21]. In contrast, *P*. *chlorantha* is mainly pollinated by moths belonging to the family of *Noctuidae*. It has a wider stigma, the distance between the viscidia is larger and the anther pockets stand strongly divergent. The pollinium has a relatively long caudicle and the large distance between the viscidia probably represents an adaptation to the distance between the pollinator’s eyes to which the viscidia are mounted [21]. Generally, hybrids and backcross individuals exhibit various intermediate features between the two parental species [21]. However, the resulting morphology generally results in limited pollination, since the pollinia are attached to the hairy zone between the eyes and the proboscis, where they cannot adhere properly and soon after uptake fall of the pollinator’s head [21]. This aspect has been shown to constitute an effective pre-zygotic barrier to cross-pollination [21].

### Sampling Sites

We investigated pure and mixed populations of *P*. *bifolia* and *P*. *chlorantha* in six distinct zones located in south of Belgium (Wallonia). Pure populations of both *Platanthera* species occurred in habitats that were characterized by pronounced differences in local growth conditions. Pure populations of *P*. *bifolia* were sampled in the natural regions of Famenne (Navaugle) and Ardennes (Saint-Hubert), both on semi-wet meadows blended with acidic soil, while pure *P*. *chlorantha* populations were examined in the Ardennes (Transinne) and Calestienne regions (Tiennes des Vignes), respectively on a semi-wet and calcareous meadow. Two mixed populations with both *Platanthera* species and intermediate plants were investigated on a calcareous grassland situated in Calestienne (Botton). Most populations were located in either Walloon public nature reserves, or private nature reserves with agreement from the Walloon Region of Belgium. Permissions to operate in those areas were obtained from the Walloon Department of Nature and Forests. Two of the populations were located on privately owned land, for which permission was granted from the owners.

All field data were collected during the flowering season (June 2014). A total of 47 individuals were haphazardly selected for each species and small parts of the roots were cut for mycorrhizal analysis (Table 1). Two main discriminating factors were used to identify *P*. *bifolia*, *P*. *chlorantha* and intermediate individuals: the length of the caudicles (mm) and the distance between the viscidia (mm) [21,22,24,38,39], which were measured according to criteria described by Nilsson [21,22]. Additionally, in each population three 1 × 1m plots were established in close proximity to the plants from which roots were sampled. In each plot, 10 topsoil samples were randomly taken with a 2.5-cm-diameter soil auger to a depth of 5 cm below the litter layer. Samples from each plot were bulked and stored in a watertight bag in a refrigerator at 5°C until processing (two weeks later). At the same time, percentage soil moisture was determined using a hand-held Hydrosense Soil Water Content Measure System in exactly the same plots where the soil samples were taken.

**Table 1:** Number of plants sampled in the different populations, with characteristics of the soil at each site.

Type*	Population	Species	Individuals sampled	Soil moisture content (%)	OM (%)	pH	P (mg/kg soil)	NO_3_^-^ (mg/kg soil)	NH_4_^+^ (mg/kg soil)
**Pure**	Transinne	*P*. *chlorantha*	5	0.36 ± 0.04	0.13 ± 0.01	5.80 ± 0.27	10.38 ± 1.36	2.78 ± 1.71	8.25 ± 1.82
**Pure**	Tienne des Vignes	*P*. *chlorantha*	5	0.27 ± 0.01	0.16 ± 0.01	6.82 ± 0.32	7.82 ± 1.04	2.15 ± 0.18	3.40 ± 1.51
**Mixed**	Botton centre	*P*. *bifolia*	5	0.24 ± 0.02	0.17 ± 0.01	8.20 ± 0.02	5.89 ± 0.54	6.82 ± 1.91	2.80 ± 2.04
		Intermediate	5	0.30 ± 0.03	0.17 ± 0.02	8.21 ± 0.05	4.73 ± 0.89	3.07 ± 0.56	0.65 ± 0.33
		*P*. *chlorantha*	5	0.24 ± 0.05	0.18 ± 0.05	8.32 ± 0.13	5.93 ± 2.04	2.07 ± 0.72	1.79 ± 0.35
**Mixed**	Botton east	*P*. *bifolia*	5	0.28 ± 0.04	0.15 ± 0.01	8.22 ± 0.09	5.52 ± 1.13	2.53 ± 0.43	0.12 ± 0.10
		Intermediate	5	0.24 ± 0.02	0.16 ± 0.02	8.26 ± 0.16	4.71 ± 0.89	1.84 ± 0.89	0.19 ± 0.13
		*P*. *chlorantha*	4	0.31 ± 0.03	0.15 ± 0.01	8.21 ± 0.13	5.28 ± 1.16	1.83 ± 1.11	0.16 ± 0.14
**Pure**	Navaugle	*P*. *bifolia*	3	0.29 ± 0.09	0.16 ± 0.04	5.56 ± 0.06	10.13 ± 3.64	0.47 ± 0.72	4.21 ± 1.55
**Pure**	Saint Hubert	*P*. *bifolia*	5	0.33 ± 0.05	0.09 ± 0.03	5.61 ± 0.18	7.44 ± 1.43	0.96 ± 0.26	2.74 ± 1.77
	Total		47						

* Pure (allopatric) populations denotes sites where only one taxon is present, whereas in mixed (sympatric) sites both species and intermediate individuals are present.

### Molecular Analysis

Amplicon libraries were created using the broad-spectrum internal transcribed spacer (ITS) primers ITS3 (5′-GCATCGATGAAGAACGCAG-3’) and ITS4OF (5′- TTACTAGGGGAATCCTTGTT-3′) [40,41]. This primer pair has been shown to be able to produce a large number of sequences and to detect a variety of orchid-associating mycorrhizal families [42]. All samples were assigned unique MID (Multiplex Identifier) barcode sequences according to the guidelines for 454 GS-FLX Lib-L amplicon sequencing (Roche Applied Science, Mannheim, Germany). Polymerase chain reaction (PCR) amplification was performed in duplicate in a 25 μl reaction volume containing 0.15 mM of each dNTP, 0.5 μM of each primer, 1 U Titanium Taq DNA polymerase, 1X Titanium Taq PCR buffer (Clontech Laboratories, Palo Alto, CA, USA), and 1 μl of a 10-times diluted DNA extract. PCR conditions were as follows: initial denaturation of 2 min at 94°C followed by 30 cycles of 45 s at 94°C, 45 s at 59°C, and 45 s at 72°C. After resolving the amplicons by agarose gel electrophoresis, amplicons within the appropriate size range (~250–500 bp) were cut from the gel and purified using the Qiaquick gel extraction kit (Qiagen, Hamburg, Germany). Purified dsDNA amplicons were quantified using the Qubit fluorometer (Invitrogen) and pooled in equimolar quantities of 1.00E+10 molecules per sample, resulting in two amplicon libraries, each representing one of the two PCR replicates. The quality of the amplicon libraries was assessed using an Agilent Bioanalyzer 2100 and high sensitivity DNA chip (Agilent Technologies, Waldbronn, Germany). Each amplicon library was loaded onto 1/8^th^ of a 454 Pico Titer Plate (PTP). Pyrosequencing was performed using the Roche GS FLX instrument and Titanium chemistry according to the manufacturer’s instructions (Roche Applied Science, Mannheim, Germany).

### Soil Analysis

Detailed soil chemical analyses were conducted using similar methods outlined in [10]. First, samples were thoroughly homogenized prior to analysis. Soil organic content was determined by percentage weight lost after combustion in a muffle oven, and soil pH was determined using a glass electrode. Soil extractable N was determined using a 1 M KCl-extraction of NH4^+^ and NO3^-^ and subsequent colorimetrical analysis using a segmented autoflow analyser [43] (Skalar, Breda, The Netherlands). Finally, soil extractable P was determined using Olson-P extraction and the extracts were colorimetrically analysed using the molybdenum blue method [44].

### Data Analysis

#### Fungal diversity

Sequences obtained from the 454 pyrosequencing run were assigned to the appropriate sample based on both barcode and primer sequences, allowing zero discrepancies, and were subsequently trimmed from the barcodes and primers using CUTADAPT 1.0 [45]. Sequences were trimmed based on a minimum Phred score of 30 (base call accuracy of 99.9%) averaged over a 50 bp moving window and sequences with ambiguous base calls or homopolymers longer than eight nucleotides were rejected, as were chimeric sequences detected by the UCHIME chimera detection program (*de novo* algorithm) [46]. Sequences which passed all quality control procedures were used as the basis for all further analyses. For further analysis, sequence data obtained for both PCR replicates were combined for each sample.

Operational Taxonomic Units (OTUs) were determined using UPARSE [47], wherein sequences exceeding 97% sequence homology were clustered into the same OTU. OTUs representing only one sequence in the whole dataset (global singletons) were removed from further analysis as it has been shown that this improves the accuracy of diversity estimates [42,48]. The remaining OTUs were assigned taxonomic identities to the highest taxonomic rank possible/family level based on BLAST [49] results of representative sequences (as indicated by UPARSE) using GenBank [50], including uncultured/environmental entries. Finally, OTUs were manually screened for possible orchid-associating mycorrhizal families based on the data provided in Table 12.1 in [51] and information of previously isolated mycorrhizal fungi from the roots, germinating seeds and protocorms of various European terrestrial orchid species that occur in similar habitats [52–55]. Only OTUs corresponding to known orchid-associating mycorrhizal families were retained for further analysis. For each taxon, total mycorrhizal fungal diversity was assessed and compared between allopatric and sympatric populations.

#### Community composition

Based on presence—absence data of the observed orchid mycorrhizal fungi in each of the sampled individuals, the fungal community composition associating with the different orchid taxa was visualized by non-metric multidimensional scaling (NMDS) using the R software package vegan [56]. Permutational analysis of variance (permanova) [57] was performed using the adonis function in the vegan package [56] to test the hypothesis that the mycorrhizal communities differed between *P*. *bifolia*, *P*. *chlorantha* and intermediate individuals. In the case of significant differences, a species-label reallocation scheme using the multiple response permutation procedures (MRPP) [58] test was implemented and pairwise comparisons were performed to see whether fungal composition differed between taxa. Finally, we used Species Indicator Analysis to investigate whether we could identify mycorrhizal fungi that were significantly associated with one of the investigated species. The multipatt function in the R package indicspecies was used to define indicator species of both individual species and combinations of species.

## Results

The quality-filtered pyrosequencing data set comprised 768 OTUs (78640 sequences), of which 93 (47615 sequences– 60.6%) were assigned to putatively orchid mycorrhizal OTUs according to Dearnaley *et al*. [51] and information from previous studies that isolated mycorrhizal fungi from the roots of *Platanthera* and related orchid species (S1 Appendix). Representative sequences for each mycorrhizal OTU found in this study were submitted in GenBank under the Accession Numbers KX776481 through KX777248.

**Fig 1:**
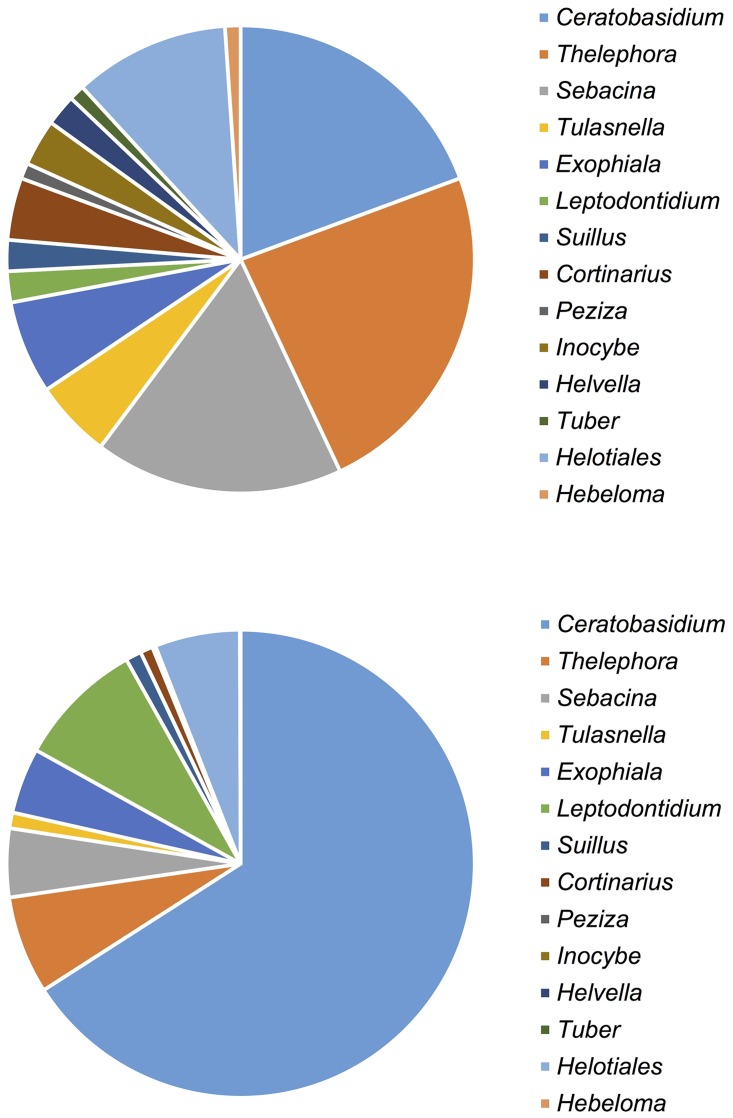
Frequency distribution displaying a) the number of mycorrhizal OTUs and b) the number of sequences across the different fungal genera that were detected on the roots of *Platanthera bifolia*, *P*. *chlorantha* and individuals displaying intermediate characteristics.

The mycorrhizal communities associating with the investigated *Platanthera* populations were clearly dominated by fungi belonging to the Ceratobasidiaceae (18 OTUs -31388 sequences) and to a lesser extent by members of the Sebacinaceae (16 OTUs– 2262 sequences) and Tulasnellaceae (5 OTUs– 517 sequences) (Fig 1a and 1b). Ectomycorrhizal fungi belonging to the Thelephoraceae were also frequently observed (22 OTUs– 3223 sequences). Besides, a large number of other ectomycorrhizal taxa known to associate with *Platanthera* and related species (*Cephalanthera*) were detected, including *Exophiala* (6 OTUs– 2165 sequences), *Cortinariu*s (4 OTUs– 413 sequences), *Inocybe* (3 OTUs– 30 sequences), *Leptodontidium* (2 OTUs—4194), *Suillus* (2 OTUs– 519 sequences), *Helvella* (2 OTUs– 10 sequences), *Tuber* (1 OTU– 22 sequences), *Peziza* (1 OTUs), and *Hebeloma* (1 OTU– 24 sequences). Finally, several members of the Helotiales were also frequently observed (10 OTUs– 2803 sequences) (Fig 1a and 1b).

**Fig 2:**
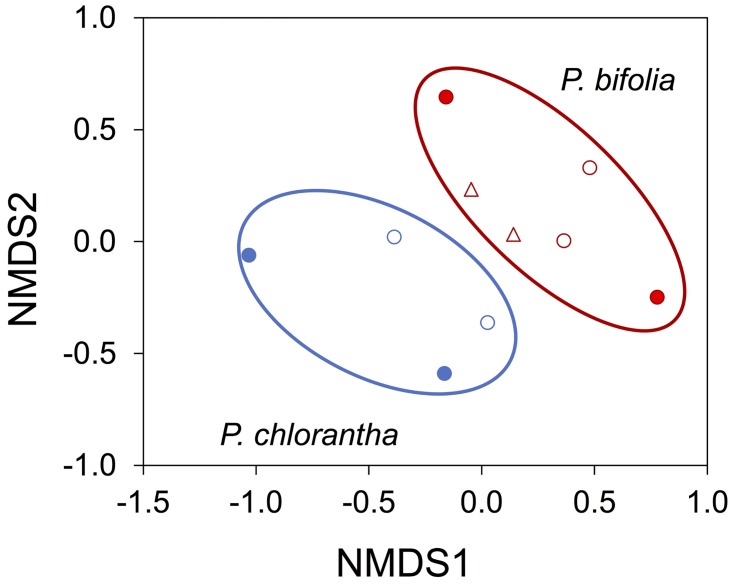
A nonmetric multidimensional scaling (nMDS) plot of mycorrhizal communities detected between pure (closed symbols) and mixed populations of *Platanthera chlorantha* (blue) and *P*. *bifolia* (red). Individuals with intermediate characteristics (open triangles) clustered within the group of *P*. *bifolia* populations. See Table 1 for identity of the populations.

In total, 69 putative orchid mycorrhizal OTUs were found in the roots of *P*. *bifolia*, 52 OTUs in the roots of *P*. *chlorantha* and 48 OTUs in the roots of individuals with intermediate characteristics. The three taxa shared 27 different mycorrhizal OTUs. Forty OTUs were shared between *P*. *bifolia* and *P*. *chlorantha*. Individuals with intermediate characteristics shared 34 and 29 OTUs with *P*. *bifolia* and *P*. *chlorantha*, respectively. Five *Ceratobasidium* strains were exclusively found in *P*. *chlorantha*, whereas *P*. *bifolia* associated with two unique strains. No *Ceratobasidium* OTU was found that uniquely occurred in individuals with intermediate morphology. Despite the substantial overlap in mycorrhizal partners, the NMDS ordination provided evidence for distinctive mycorrhizal communities associating with *P*. *bifolia* and *P*. *chlorantha* (Fig 2), especially between pure populations, but less between the mixed populations. The ordination represented the data structure well (stress = 0.13). Individuals with intermediate characteristics clustered together with *P*. *bifolia* individuals. Analysis by permanova confirmed that community composition of mycorrhizal fungi was significantly different between the three sets of plants (*P*. *bifolia*, *P*. *chlorantha* and intermediates). Pairwise comparisons using the MRPP procedure showed that community composition was significantly different between *P*. *bifolia* and *P*. *chlorantha*, but not between *P*. *bifolia* and individuals with intermediate characteristics. Finally, Species Indicator Analysis did not reveal any fungal OTUs that were significantly associated with one of the investigated species

Soil pH varied considerably between populations, ranging from 5.56 to 8.32 (average ± SD: 7.32 ± 1.32) (Table 1). Soil extractable nutrients also varied substantially between populations, constituting a gradient in soil N (NH4^+^ + NO3^-^) ranging from 1.83 mg N/kg soil to 11.03 mg N/kg soil (average 4.86 mg N/kg soil, SD 3.10), whereas soil P varied between 4.71 mg P/kg soil and 10.38 mg P/kg soil (average 6.78 mg P/kg soil, SD 2.09). Percentage soil moisture varied between 24.2% and 36.5%, whereas percentage organic content varied between 9.2% and 18.2%. Despite the observed differences in soil nutrient content between sites, there was no correlation between soil nutrients and pH and mycorrhizal community composition, but partial CCA analysis showed that mycorrhizal communities were significantly (pseudo-*F* = 1.31, *P* = 0.02) related to soil moisture content.

## Discussion

### Fungal Diversity and Specificity

In this study, we applied 454 amplicon pyrosequencing to assess the mycorrhizal communities associating with the closely related terrestrial orchids *Platanthera bifolia* and *P*. *chlorantha* and some individuals with intermediate morphology. Our results clearly illustrated that the dominant fungi associating with both *Platanthera* species were members of the Ceratobasidiaceae, although several representatives of the Tulasnellaceae and Sebacinales were detected as well, albeit at minor frequencies. These results largely confirm previous studies that have shown that species of the genus *Platanthera* mainly associate with fungi of the Ceratobasidiaceae [59–64]. These results are also in line with in vitro seed germination experiments. Using several different *Ceratobasidium* and *Tulasnella* strains, Rasmussen [65] showed that seed germination percentages were generally highest (up to 80%) when strains from *Ceratobasidium* were used. In contrast, strains of *Tulasnella* resulted in seed germination percentages < 40%. On the other hand, strains of *Tulasnella* generally led to better seedling growth than *Ceratobasidium* strains [65]. These observations suggest that strains of different fungal families may be involved in the seed germination process of *Platanthera*. Our results are in line with these findings, as representative strains of both fungal families were found associating with the studied *Platanthera* species and are consistent with observations made in other orchid species that use different mycorrhizal fungi for different phases of the plant life cycle [65].

Besides fungal strains from the Ceratobasidiaceae and Tulasnellaceae, members of the Thelephoraceae were observed as well. These fungi have been frequently shown to associate with several species of other European orchid genera, including *Cephalanthera*, [52], *Neottia* [10] or *Epipactis* [55]. These orchid species often grow in closed forest habitats with limited light availability and associate with fungi that usually form ectomycorrhizae on surrounding trees [52]. In the forest orchids *Cephalanthera damasonium* and *C*. *longifolia*, several members of the Thelephoraceae, including *Tomentella* and *Pseudotomentella*, were also found in germinating seeds [66], suggesting that these fungi may promote seed germination. However, whether the observed ectomycorrhizal fungi actually stimulate germination or contribute to the nutrient budget of *Platanthera* warrants further investigation. Previous research using stable isotopes has shown that plants of *P*. *chlorantha* were not significantly enriched in nitrogen or carbon [52], suggesting that ectomycorrhizal fungi may not be not truly orchid mycorrhizal in *Platanthera*. Interestingly, we also observed the frequent occurrence of *Leptodontidium orchidicola*. Although it is not entirely clear whether this heliotelean ascomycete is genuinely mycorrhizal [67], it has been observed in *P*. *chlorantha* before [52] as well as in other *Platanthera* species (e.g. *P*. *pollostantha*, *P*. *orbiculata*, *P*. *hyperborea*) [59,64,68].

In common with other European terrestrial orchids, individual plants were associated with several fungi simultaneously [53,54,69]. The function of sharing multiple fungal partners in orchids is poorly understood. Previous research has shown that orchids often use different mycorrhizal fungi for different phases of the plant life cycle [65], and it is likely that some of the early mycorrhizal fungi remain present in the orchid’s roots, without fulfilling a proper role anymore. Jacquemyn *et al*. [54] hypothesized that under nutrient-poor conditions, sharing a diverse range of mycorrhizal fungal symbionts could optimize the access of the plant to its growth-limiting resources. Furthermore, during a stress condition, as diverse fungi are likely to play a different role in the acquisition of nutrient resources [13,70], the ability of the plant to switch from different fungal partners may increase nutrient uptake and the probability of the plant to survive. However, to gain a better knowledge of these physiological mechanisms, more detailed investigations are needed.

### Mycorrhizal Divergence

Despite the substantial overlap in mycorrhizal partners, the NMDS analysis provided evidence for distinctive mycorrhizal communities associating with *P*. *bifolia* and *P*. *chlorantha*, particularly in pure populations. In mixed populations, i.e. at sites where the two species co-occurred, the differences were less pronounced, but still there was a clear difference between *P*. *bifolia* and *P*. *chlorantha*. Individuals with intermediate morphology clustered within *P*. *bifolia*, indicating that their mycorrhizal profiles were similar. The pronounced differences between pure populations are most likely related to differences in local soil conditions. Pure populations of *P*. *bifolia* were sampled on wet, somewhat acidic soils, whereas *P*. *chlorantha* was sampled on more calcareous-rich soils. Previous research has shown that local environmental conditions can have a significant impact on orchid mycorrhizal communities. For example, Oja *et al*. [11] sampled populations of the terrestrial orchid *Neottia ovata* from forest and meadows and showed significant differences in mycorrhizal communities between these habitats. Similarly, Pandey *et al*. [9] and Jacquemyn *et al*. [10] showed that mycorrhizal communities varied between populations of *Piperia yadonii* and *Neottia ovata*, respectively, and that differences in community composition were related to local environmental conditions. In *N*. *ovata*, for example, differences in community composition were mainly due to differences in soil moisture content and pH [10]. In *Platanthera*, mycorrhizal communities were affected by soil moisture content as well, but not by pH. However, this was mainly due to the fact that the two species responded differently to varying pH conditions, suggesting that the relationship between environmental conditions and mycorrhizal communities depends on orchid species. Clearly, more populations need to be sampled to firmly establish the relationship between mycorrhizal communities and edaphic conditions in orchids.

In the two studied mixed populations, where differences in edaphic conditions were less pronounced, communities associating with *P*. *chlorantha* were still significantly different from those associating with *P*. *bifolia*. These results are in line with previous observations, which have shown that co-occurring orchids may often be associated with different communities of mycorrhizal fungal symbionts [13,17,20,71] and therefore may constitute an important mechanism contributing to coexistence of orchids. For example, Waud *et al*. [8] recently showed that three co-occurring meadow species (*Orchis mascula*, *Anacamptis morio* and *Gymnadenia conopsea*) showed significantly different mycorrhizal communities. These differences, in turn, affected spatial patterns of seed germination and above-ground distribution of adult plants [17]. Similarly, in a hybrid zone of three *Orchis* species, fungal community composition differed significantly between the three pure species, which also occupied different locations in the population [20]. Interestingly, hybrids between *O*. *purpurea* and *O*. *militaris* showed a mycorrhizal community composition that was similar to that of *O*. *purpurea*. In the case of *Platanthera*, individuals with intermediate morphology showed mycorrhizal communities similar to those of *P*. *bifolia*. Although the exact processes leading to the observed variation in floral morphology are still unclear, molecular analyses and analyses of floral scent profiles have shown that these individuals with intermediate morphology were most likely not hybrids between *P*. *bifolia* and *P*. *chlorantha*, but pure *P*. *bifolia* [27]. Our results are in line with these observations.

### Implications for Maintenance of Species Integrity

Previous research has shown that several pre-zygotic barriers (phenological isolation, pollinator specialization, mechanical isolation) contribute to reproductive isolation acting between *P*. *bifolia* and *P*. *chlorantha* [46]. However, the possibility that mycorrhizal divergence has contributed to reproductive isolation has not been considered. Mycorrhizal divergence may contribute to reproductive isolation in two different ways. First, differences in mycorrhizal communities may lead to reduced seed, seedling or adult fitness in heterospecific habitat so that populations of both species occur in disparate geographical regions and chances for gene flow by pollen or seeds between species are restricted (i.e. ecogeographic isolation). Second, the mycorrhizal communities differ between the two species to such an extent that hybrid seeds are less likely to germinate and survive than parental seeds (hybrid inviability).

In this study we have shown that the two studied *Platanthera* species associated with distinctive mycorrhizal communities, but that differences in mycorrhizal communities were not strong enough to create eco-geographic isolation. Moreover, our results showed that individuals with intermediate morphological characters not only had the same genetic architecture and the same floral scent as *P*. *bifolia* [27], but also displayed a similar mycorrhizal community composition, suggesting that hybridization in both populations was limited or absent. It is therefore tempting to suggest that the observed differences in mycorrhizal associations between species led to an effective barrier to hybridization due to incompatibilities between orchid seeds and mycorrhizal fungi and therefore contribute to reproductive isolation. However, differences in mycorrhizal communities between the two species were smaller in mixed than in pure populations and the two species shared a considerable number of fungal strains, suggesting that mycorrhizal fungi are most likely not directly involved in maintaining species boundaries between the two *Platanthera* species. To prove that mycorrhizal fungi are effectively involved in contributing to reproductive isolation, future research should perform seed germination experiments using both pure and hybrid seeds and assess seed germination and protocorm formation at different locations within pure and mixed populations.

## Supporting Information

S1 Appendix List of operational taxonomic units (OTUs) corresponding to orchid-associating mycorrhizal families discovered in this study. (DOCX)
