# Supplementary material for: Mycorrhizal Fungal Diversity and Community Composition in Two Closely Related Platanthera (Orchidaceae) Species
Source: PLoS One. 2016 Oct 3;11(10):e0164108. doi: 10.1371/journal.pone.0164108 (PMC5047478; doi:10.1371/journal.pone.0164108)
Supplement: S1 Appendix — (DOCX) [file pone.0164108.s001.docx]

**Appendix 1** List of operational taxonomic units (OTUs) corresponding to orchid-associating mycorrhizal families discovered in this study.

| **OTU** | **# sequences** | **Bit score S'** | **Expected** | **Identities** | **Length** | **Closest match** |
| --- | --- | --- | --- | --- | --- | --- |
| OTU1 | 10696 | 457.243 | 4.1E-125 | 249 | 250 | Uncultured *Ceratobasidium* clone |
| OTU3 | 7247 | 451.703 | 1.9E-123 | 248 | 250 | *Thanatephorus fusisporus* voucher KC833 18S |
| OTU4 | 4729 | 462.783 | 8.9E-127 | 250 | 250 | Ceratobasidiaceae sp. CBS 499.93 5.8S |
| OTU9 | 4003 | 462.783 | 8.9E-127 | 250 | 250 | *Leptodontidium* sp. nwa_besc_80cb |
| OTU118 | 3195 | 422.156 | 1.5E-114 | 243 | 250 | Uncultured *Ceratobasidium* clone SsA |
| OTU8 | 1972 | 462.783 | 8.9E-127 | 250 | 250 | *Exophiala* sp. nwa_sqmc_26_5e |
| OTU11 | 1841 | 435.083 | 1.9E-118 | 245 | 250 | Ceratobasidiaceae sp. CBS 501.93 5.8S |
| OTU6 | 1740 | 416.616 | 7E-113 | 242 | 250 | Uncultured *Tomentella* clone 113eR.AZ 18S |
| OTU10 | 1250 | 340.904 | 4.35E-90 | 232 | 252 | *Ceratobasidium* sp. UAMH 9847 |
| OTU12 | 996 | 462.783 | 8.9E-127 | 250 | 250 | *Sebacina* sp. LM5274 |
| OTU14 | 990 | 462.783 | 8.9E-127 | 250 | 250 | Uncultured Helotiales clone 53 |
| OTU15 | 798 | 462.783 | 8.9E-127 | 250 | 250 | *Ceratobasidium albasitensis* |
| OTU28 | 787 | 444.316 | 3.2E-121 | 247 | 250 | Ceratobasidiaceae sp. CBS 502.93 |
| OTU13 | 672 | 462.783 | 8.9E-127 | 250 | 250 | Helotiales sp. REF046 |
| OTU30 | 545 | 462.783 | 8.9E-127 | 250 | 250 | *Sebacina epigaea* voucher TUB 020017 |
| OTU25 | 500 | 462.783 | 8.9E-127 | 250 | 250 | Helotiales sp. X19 |
| OTU17 | 497 | 418.463 | 2E-113 | 242 | 250 | *Ceratobasidium* sp. JTO-2010a |
| OTU23 | 475 | 462.783 | 8.9E-127 | 250 | 250 | Uncultured Tulasnellaceae clone OmiAB03_B_21 |
| OTU22 | 460 | 372.297 | 1.5E-99 | 234 | 250 | Uncultured *Sebacina* isolate B96_2 |
| OTU19 | 440 | 462.783 | 8.9E-127 | 250 | 250 | *Suillus luteus* isolate N3 |
| OTU31 | 404 | 457.243 | 4.1E-125 | 249 | 250 | Uncultured *Tomentella* clone VBM2a-09 |
| OTU40 | 269 | 462.783 | 8.9E-127 | 250 | 250 | Uncultured *Tomentella* isolate UE_ITA003 |
| OTU43 | 239 | 457.243 | 4.1E-125 | 249 | 250 | Uncultured Helotiales clone d153b_4_10 |
| OTU47 | 226 | 457.243 | 4.1E-125 | 249 | 250 | Uncultured *Tomentella* clone SC_ITS_087 |
| OTU746 | 191 | 424.003 | 4.2E-115 | 244 | 251 | *Leptodontidium orchidicola* |
| OTU84 | 173 | 462.783 | 8.9E-127 | 250 | 250 | Uncultured Cortinarius clone 3065AM |
| OTU104 | 154 | 462.783 | 8.9E-127 | 250 | 250 | *Cladophialophora chaetospira* strain CBS 491.70 |
| OTU41 | 149 | 440.623 | 4.2E-120 | 247 | 251 | Uncultured Thelephoraceae clone PP_D3_1_242_3 |
| OTU64 | 123 | 457.243 | 4.1E-125 | 250 | 251 | Uncultured Ceratobasidium clone OF2a |
| OTU70 | 106 | 416.616 | 7E-113 | 250 | 260 | Uncultured Thelephoraceae clone OTUrs15 |
| OTU65 | 105 | 451.703 | 1.9E-123 | 248 | 250 | Uncultured Helotiales clone G4c890H |
| OTU73 | 104 | 451.703 | 1.9E-123 | 249 | 251 | Helotiales sp. 16 MV-2011 strain PA 070 |
| OTU101 | 88 | 457.243 | 4.1E-125 | 249 | 250 | Uncultured Thelephoraceae OTU: SNG.82 18S |
| OTU111 | 86 | 457.243 | 4.1E-125 | 250 | 251 | Uncultured Sebacinaceae clone SC_ITS_114 |
| OTU117 | 82 | 462.783 | 8.9E-127 | 250 | 250 | *Ceratobasidium* sp. Rr82 |
| OTU89 | 79 | 462.783 | 8.9E-127 | 250 | 250 | *Suillus granulatus* isolate |
| OTU87 | 77 | 462.783 | 8.9E-127 | 250 | 250 | *Sebacina dimitica* strain TUB 019989 |
| OTU103 | 72 | 462.783 | 8.9E-127 | 250 | 250 | Uncultured *Cortinarius* clone DH_D5_1 |
| OTU92 | 71 | 457.243 | 4.1E-125 | 250 | 251 | Uncultured ectomycorrhiza (Ascomycota) isolate |
| OTU96 | 66 | 444.316 | 3.2E-121 | 247 | 250 | Uncultured Helotiales clone PA K3 02 |
| 0TU124 | 63 | 462.783 | 8.9E-127 | 250 | 250 | Uncultured ectomycorrhizal fungus clone |
| OTU195 | 59 | 457.243 | 4.1E-125 | 249 | 250 | Uncultured Ceratobasidiaceae clone OTU-3-28 |
| OTU132 | 54 | 429.543 | 9E-117 | 244 | 250 | Uncultured Ceratobasidiaceae isolate 978 |
| OTU105 | 51 | 462.783 | 8.9E-127 | 250 | 250 | Uncultured Helotiales clone LP110074 |
| OTU110 | 45 | 462.783 | 8.9E-127 | 250 | 250 | Uncultured Pezizales clone 19-184-1z |
| OTU109 | 43 | 427.696 | 3.2E-116 | 244 | 250 | Uncultured Helotiales genomic DNA |
| OTU157 | 43 | 462.783 | 8.9E-127 | 250 | 250 | Uncultured *Tomentella* isolate 4.8137.1.D |
| OTU174 | 39 | 457.243 | 4.1E-125 | 249 | 250 | Uncultured ectomycorrhiza (*Tomentella*) isolate 5i1 |
| OTU146 | 33 | 462.783 | 8.9E-127 | 250 | 250 | Uncultured Helotiales genomic DNA |
| OTU206 | 31 | 462.783 | 8.9E-127 | 250 | 250 | Uncultured Thelephoraceae genomic DNA |
| OTU488 | 30 | 462.783 | 8.9E-127 | 250 | 250 | *Exophiala* sp. bc_besc_86f |
| OTU651 | 26 | 433.236 | 7E-118 | 245 | 250 | *Exophiala* sp. KL-2011f strain CBS 122270 |
| OTU191 | 24 | 462.783 | 8.9E-127 | 250 | 250 | *Hebeloma sinapizans* genomic DNA |
| OTU210 | 23 | 440.623 | 4.2E-120 | 246 | 250 | Uncultured fungus genomic DNA sequence |
| OTU270 | 23 | 412.923 | 9.1E-112 | 241 | 250 | Uncultured ectomycorrhiza (Thelephoraceae) clone L6CE4 |
| OTU159 | 22 | 462.783 | 8.9E-127 | 250 | 250 | *Tuber anniae* voucher KUN:F59232 |
| OTU205 | 22 | 455.396 | 1.5E-124 | 248 | 250 | Uncultured *Sebacina* mycobiont of *Dipsacus sylvestris* clone 6872 |
| OTU409 | 19 | 457.243 | 4.1E-125 | 249 | 250 | *Inocybe melliolens* voucher EL224_06 |
| OTU346 | 18 | 446.163 | 9E-122 | 247 | 250 | Uncultured ectomycorrhiza (Thelephoraceae) |
| OTU343 | 17 | 429.543 | 9E-117 | 244 | 250 | Uncultured Tulasnellaceae clone OTUA2_PLATEAGA_6_A3 |
| OTU218 | 14 | 414.77 | 2.5E-112 | 242 | 250 | Uncultured *Cortinarius* clone MT2 |
| OTU219 | 13 | 462.783 | 8.9E-127 | 250 | 250 | Uncultured *Tomentella* clone 3113/1N |
| OTU299 | 13 | 451.703 | 1.9E-123 | 248 | 250 | Uncultured *Sebacina* clone 10361 |
| OTU484 | 13 | 462.783 | 8.9E-127 | 250 | 250 | Uncultured *Sebacina* isolate TUB 019456 clone G3 |
| OTU216 | 12 | 462.783 | 8.9E-127 | 250 | 250 | Uncultured ectomycorrhiza (*Tomentella*) morphotype 19 |
| OTU287 | 12 | 457.243 | 4.1E-125 | 249 | 250 | Uncultured *Tomentella* clone M5MPC1 |
| OTU234 | 11 | 401.843 | 2E-108 | 240 | 251 | Uncultured *Sebacina* mycobiont of *Dipsacus sylvestris* clone 6872 |
| OTU288 | 11 | 351.984 | 2.01E-93 | 234 | 254 | Uncultured Sebacinaceae clone OTU-0624 |
| OTU413 | 11 | 429.543 | 9E-117 | 244 | 250 | Uncultured Tulasnellaceae clone J1M-2A |
| OTU465 | 10 | 451.703 | 1.9E-123 | 248 | 250 | *Ceratobasidium* sp. AG-I |
| OTU286 | 9 | 462.783 | 8.9E-127 | 250 | 250 | Uncultured Sebacinaceae clone OTU-0439 |
| OTU347 | 8 | 462.783 | 8.9E-127 | 250 | 250 | *Tomentella* sp. LM4990 |
| OTU414 | 8 | 165.472 | 2.81E-37 | 200 | 252 | *Tomentella* sp. 4 CG-2012 |
| OTU503 | 8 | 462.783 | 8.9E-127 | 250 | 250 | *Rhizoctonia solani* isolate Y2 |
| OTU418 | 7 | 436.93 | 5.4E-119 | 246 | 250 | Uncultured Tulasnellaceae isolate 479 |
| OTU451 | 7 | 418.463 | 2E-113 | 242 | 250 | Uncultured mycorrhiza (Tulasnellaceae) 4096 |
| OTU582 | 7 | 307.664 | 4.41E-80 | 224 | 252 | Uncultured Sebacinaceae clone OTU-3-77 |
| OTU442 | 6 | 462.783 | 8.9E-127 | 250 | 250 | *Rhizoctonia* sp. AG-C isolate WUF-ST-RhT4-7 |
| OTU448 | 6 | 462.783 | 8.9E-127 | 250 | 250 | *Helvella lacunosa* voucher F 1187326 |
| OTU460 | 6 | 364.91 | 2.58E-97 | 233 | 250 | Uncultured *Inocybe* clone NAATOTU146 |
| OTU415 | 5 | 462.783 | 8.9E-127 | 250 | 250 | Uncultured Thelephoraceae clone OTU-3-31 |
| OTU637 | 5 | 451.703 | 1.9E-123 | 249 | 251 | *Inocybe nitidiuscula* voucher UBC F18170 |
| OTU590 | 4 | 414.77 | 2.5E-112 | 245 | 254 | Uncultured *Thanatephorus* clone OTU82_HY522PD02B58D0 |
| OTU646 | 4 | 457.243 | 4.1E-125 | 249 | 250 | *Helvella* sp. UC 1999221 |
| OTU699 | 4 | 457.243 | 4.1E-125 | 249 | 250 | Uncultured *Sebacina* isolate TUB 019446 clone B3 |
| OTU522 | 3 | 237.491 | 5.87E-59 | 213 | 253 | Sebacinales sp. PC38 |
| OTU632 | 3 | 457.243 | 4.1E-125 | 249 | 250 | *Exophiala* sp. SST-2011 voucher CBS:121843 |
| OTU657 | 3 | 451.703 | 1.9E-123 | 248 | 250 | Uncultured Sebacinales clone 6586 |
| OTU650 | 2 | 457.243 | 4.1E-125 | 249 | 250 | Uncultured *Tomentella* clone 2007BBE8 |
| OTU714 | 2 | 390.763 | 4.3E-105 | 238 | 251 | Uncultured *Sebacina* isolate TUB 019313 |
| OTU745 | 2 | 446.163 | 9E-122 | 247 | 250 | *Ceratobasidium* sp. JTO-2010a clone JTO475 |
| OTU747 | 2 | 462.783 | 8.9E-127 | 250 | 250 | Uncultured *Tomentella* isolate II.8261.2 |
| OTU755 | 2 | 390.763 | 4.3E-105 | 238 | 250 | Uncultured *Ceratobasidium* clone AL4b |
